# Bis[2,6-bis­(1*H*-pyrazol-1-yl)pyridine]­deca­kis­(μ_2_-3-nitro­benzoato)bis­(3-nitro­benzoato)tetra­dysprosium(III): a linear tetra­nuclear dysprosium compound based on mixed N- and O-donor ligands

**DOI:** 10.1107/S1600536814006060

**Published:** 2014-04-02

**Authors:** Rong Hua, Xiao-Liu Wu, Jin-Ying Li

**Affiliations:** a China Institute of Atomic Energy, Beijing 102413, People’s Republic of China

## Abstract

The title compound, [Dy_4_(C_7_H_4_NO_4_)_12_(C_11_H_9_N_5_)_2_] or Dy_4_(*L*1)_12_(*L*2)_2_, where H*L*1 = 3-nitro­benzoic acid and H*L*2 = 2,6-bis­(1*H*-pyrazol-1-y1)pyridine, is a linear tetra­nuclear complex possessing inversion symmetry. The two central inversion-related Dy^III^ atoms are seven-coordinate, DyO_7_, with a monocapped triangular-prismatic geometry. The outer two Dy^III^ atoms are eight-coordinate, DyO_5_N_3_, with a bicapped triangular-prismatic geometry. The outer adjacent Dy^III^ atoms are bridged by three *L*1^−^ carboxyl­ate groups, while the inner inversion-related Dy^III^ atoms are bridged by four *L*1^−^ carboxyl­ate groups. The *L*2 ligands are terminally coordinated to the outer Dy^III^ atoms in a tridentate manner. In the crystal, mol­ecules are linked *via* C—H⋯O hydrogen bonds, forming a two-dimensional network parallel to (001). Two carboxyl­ate O atoms, and N and O atoms of three nitro groups, are disordered over two positions, with a refined occupancy ratio of 0.552 (6):0.448 (6).

## Related literature   

For background to single mol­ecular magnets, see: Zheng *et al.* (2008 ▶); Wu *et al.* (2009 ▶); Guo *et al.* (2012 ▶). For examples of multinuclear Dy-based compounds, see: Abbas *et al.* (2010 ▶); Blagg *et al.* (2011 ▶); Hussain *et al.* (2009 ▶); Tian *et al.* (2012 ▶); Xu *et al.* (2010 ▶). For details of a linear tetra­nuclear Dy^III^ complex, see: Lin *et al.* (2012 ▶).
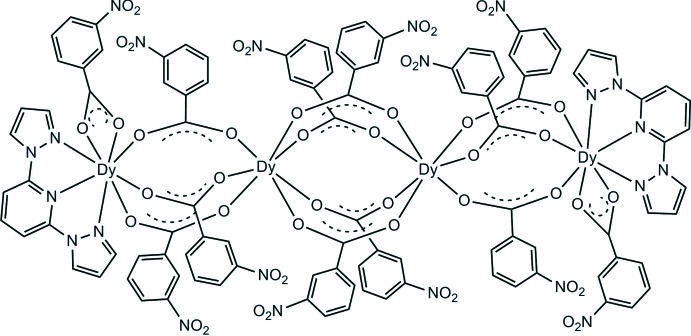


## Experimental   

### 

#### Crystal data  

[Dy_4_(C_7_H_4_NO_4_)_12_(C_11_H_9_N_5_)_2_]
*M* *_r_* = 3065.81
Triclinic, 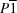

*a* = 14.2514 (3) Å
*b* = 14.9274 (5) Å
*c* = 14.9616 (3) Å
α = 100.443 (2)°
β = 111.754 (1)°
γ = 99.695 (2)°
*V* = 2808.87 (12) Å^3^
*Z* = 1
Mo *K*α radiation
μ = 2.74 mm^−1^
*T* = 296 K
0.20 × 0.20 × 0.19 mm

#### Data collection  

Bruker APEX2 CCD area-detector diffractometer
Absorption correction: multi-scan (*SADABS*; Sheldrick, 1996 ▶) *T*_min_ = 0.611, *T*_max_ = 0.624
42971 measured reflections
11020 independent reflections
9747 reflections with *I* > 2σ(*I*)
*R*_int_ = 0.025

#### Refinement  

*R*[*F* ^2^ > 2σ(*F* ^2^)] = 0.022
*wR*(*F* ^2^) = 0.058
*S* = 1.13
11020 reflections
848 parameters
24 restraints
H-atom parameters constrained
Δρ_max_ = 0.66 e Å^−3^
Δρ_min_ = −0.67 e Å^−3^

### 

Data collection: *APEX2* (Bruker, 2008 ▶); cell refinement: *SAINT* (Bruker, 2008 ▶); data reduction: *SAINT*; program(s) used to solve structure: *SHELXS97* (Sheldrick, 2008 ▶); program(s) used to refine structure: *SHELXL97* (Sheldrick, 2008 ▶); molecular graphics: *DIAMOND* (Brandenburg, 2005 ▶); software used to prepare material for publication: *SHELXL97*.

## Supplementary Material

Crystal structure: contains datablock(s) I. DOI: 10.1107/S1600536814006060/su2704sup1.cif

Structure factors: contains datablock(s) I. DOI: 10.1107/S1600536814006060/su2704Isup2.hkl

CCDC reference: 992494

Additional supporting information:  crystallographic information; 3D view; checkCIF report

## Figures and Tables

**Table 1:** Hydrogen-bond geometry (Å, °)

*D*—H⋯*A*	*D*—H	H⋯*A*	*D*⋯*A*	*D*—H⋯*A*
C10—H10⋯O22^i^	0.93	2.45	3.366 (4)	170
C43—H43⋯O2^ii^	0.93	2.47	3.212 (5)	137
C51—H51⋯O6^iii^	0.93	2.32	3.243 (5)	172

Symmetry codes: (i) 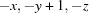; (ii) 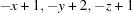; (iii) 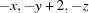.

**Fig. 1.**
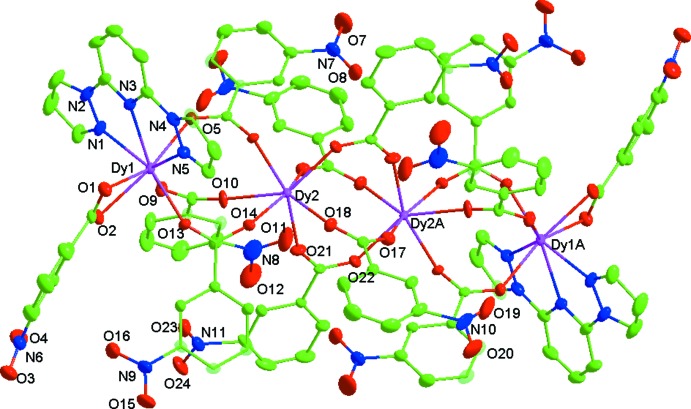
The coordination environment of the DyIII ions in the title compound, with atom labelling of heteroatoms only. Displacement ellipsoids are drawn at the 50% probability level [H atoms have been omitted for clarity; Dy/pink, C/bright green, N/blue, O/red; symmetry code: A = -x, -y+1, -z].

**Fig. 2.**
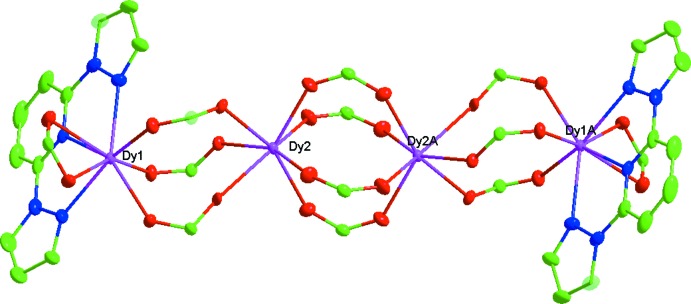
A view of the simplified tetranuclear structure of the title compound.

**Fig. 3.**
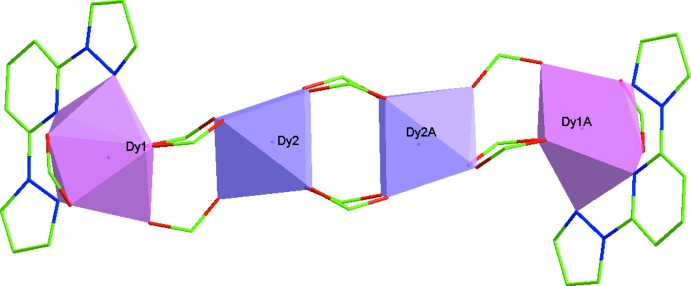
The polyhedral view of the tetranuclear structure of the title compound.

**Fig. 4.**
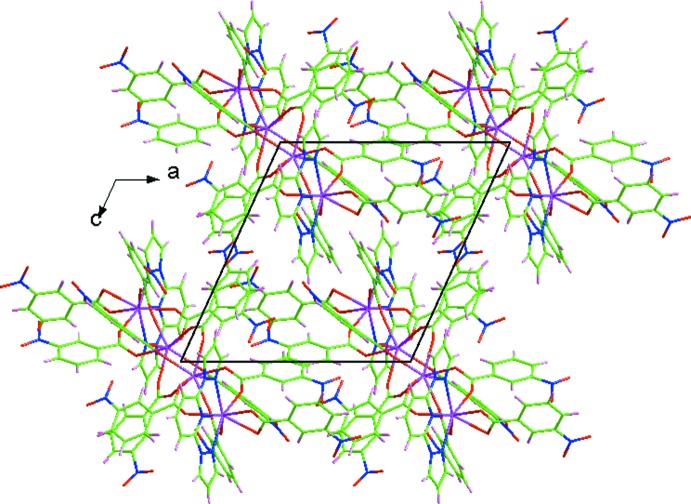
A view along the b axis of the crystal packing of the title compound [Dy/pink, C/bright green, N/blue, O/red].

## supplementary crystallographic information

### 1. Comment

Single molecule magnets (SMMs) have potential applications in data storage, quantum information processing (Zheng *et al.*, 2008), magnetic refrigeration, and high-density information storage (Wu *et al.*, 2009).SMMs are mononuclear or multinuclear transition metal-based clusters or rare-earth metal-based clusters with no interactions between molecules. Mn^II^-based SMMs were the first to be fully investigated, whereas recently 4f- or 5f-based compounds, especially Dy^III^-based compounds, have been investigated because of their potential significant magnetic anisotropy and large energy barriers (Guo *et al.*, 2012). A number of multinuclear Dy-based compounds have been synthesized, for example, Dy_2_ (Xu *et al.*, 2010), Dy_3_ (Hussain *et al.*, 2009), Dy_4_ (Abbas *et al.*, 2010), Dy_5_ (Blagg *et al.*, 2011), and Dy_8_ (Tian *et al.*, 2012). In line with our interest in designing multinuclear Dy-based compounds, we report herein on the synthesis and crystal structure of a new and novel linear tetranuclear dysprosium compound, which is based on mixed N-donor and O-donor ligands.

The molecular structure of the title compound is illustrated in Fig. 1. It possesses inversion symmetry and the asymmetric unit is composed of two crystallographic independent Dy^III^ ions, six crystallographic independent 3-nitro benzoate *L*1^-^ ligands, and one crystallographic independent 2,6-di(1*H*-pyrazol-1-y1)pyridine *L*2 ligand. The coordination spheres of the two Dy^III^ ion are different. Atom Dy1 has a DyO_5_N_3_ bi-capped triangular-prism geometry, completed by three nitrogen atoms (N1, N3, N5) from one *L*2 ligand, and five oxygen atoms (O1, O2, O5, O9, O13) from three *L*1^-^ ligands, where the capped atoms are N5, O9. Atom Dy2 has a DyO_7_ monocapped triangular-prism geometry, created by seven oxygen atoms (O6, O10, O14, O17, O18, O21, O22) from seven *L*1^-^ ligands, where the capped atom is O10. The Dy—O/N bond length are in the normal range. The *L*2 ligand coordinates to the Dy1 atoms in a tridentate manner, whereas the *L*1^-^ ligands display two kinds of coordination mode, chelate and bidentate. The chelate mode involves the outer pair of Dy atoms, while the bidentate mode involves the inversion related Dy2 atoms in the center of the tetranuclear complex.

The overall structure of the title compound is a tetranuclear cluster in the Dy1—Dy2—Dy2A—Dy1A arrangement, see Figs. 1, 2, and 3. Atoms Dy1 and Dy2 are bridged by three *L*1^-^ carboxylate ligands with a metal-to-metal distance of 4.88 (2) Å. Through inversion symmetry the Dy1—Dy2 fragment connects to the symmetry-related Dy2A···Dy1A fragment *via* the Dy2—Dy2A connection linked by four *L*1^-^ carboxyl groups. The Dy2···Dy2A distance is 4.34 (2) Å. The span of this tetranuclear cluster estimated by the distance of two terminal metal ions Dy1 and Dy1A is 14.07 (2) Å [symmetry code: A = -*x*, -*y*+1, -*z*].

In the crystal, molecules are linked via C-H···O hydrogen bonds forming a two-dimensional network parallel to (001); see Table 1 and Fig. 4.

Although the ligand 2,6-di(1*H*-pyrazol-1-y1) pyridine is extensively used to construct metal-organic compounds, it has rarely been used in combination with lanthanide ions, and there is no precedent of a cluster compound containing a lanthanide ion and the ligand 2,6-di(1*H*-pyrazol-1-y1)pyridine. The title compound is the first such compound to be synthesized and is a promising way to target multinuclear lanthanide clusters.

Moreover, in the literature, there is only one other report of a linear tetranuclear Dy^III^ complex (Lin *et al.*, 2012), [Dy_4_(*L*)_2_(C_6_H_5_COO)_12_(MeOH)_4_] (*L* = 2,6-bis((furan-2-ylmethylimino)methyl)-4-methyl-phenol), where the Dy^III^ sites are fully coordinated by O atoms, rather than being coordinated by both O and N atoms as observed in the title compound. Interestingly the metal-to-metal distances (4.24 (2) Å and 4.06 (2) Å) reported there are slightly shorter than those observed in the title compound.

### 2. Experimental

DyCl_3_ (0.2 mmol), 2,6-di(1*H*-pyrazol-1-y1)pyridine (2 mmol), nitro benzoic acid (0.8 mmol), Na_2_CO_3_ (0.4 mmol) and H_2_O (10 mL) were mixed together, then transferred into a 25 ml Teflon-lined reactor. This was heated at 483 K for 3 days. The mixture was then cooled to room temperature, at a rate of 3 K/h, and yielded block-like colourles crystals in a 58% yield based on Dy.

### 3. Refinement

The NH H atoms could be located in a difference Fourier map. The C and N-bound H atoms were included in calculated positions and treated as riding: N-H = 0.86 Å, C—H = 0.93 – 0.97 Å with *U*_iso_(H) = 1.2*U*_eq_(N/C). Two O atoms of carboxyl groups (O10/O10' and O14/O14') and atoms in three nitro groups

(N10/N10', O19/O19', O20/O20', N11/N11', O23/O23', O24/O24' and O16/O16') are disordered over two positions with a refined occupancy ratio of 0.448 (6):0.552 (6).

### Crystal data

**Table:**

[Dy_4_(C_7_H_4_NO_4_)_12_(C_11_H_9_N_5_)_2_]	*Z* = 1
*M**_r_* = 3065.81	*F*(000) = 1504
Triclinic, *P*1	*D*_x_ = 1.812 Mg m^−^^3^
*a* = 14.2514 (3) Å	Mo *K*α radiation, λ = 0.71073 Å
*b* = 14.9274 (5) Å	Cell parameters from 9289 reflections
*c* = 14.9616 (3) Å	θ = 2.6–27.7°
α = 100.443 (2)°	µ = 2.74 mm^−^^1^
β = 111.754 (1)°	*T* = 296 K
γ = 99.695 (2)°	Block, colourless
*V* = 2808.87 (12) Å^3^	0.20 × 0.20 × 0.19 mm

### Data collection

**Table:**

Bruker APEX2 CCD area-detector diffractometer	11020 independent reflections
Radiation source: fine-focus sealed tube	9747 reflections with *I* > 2σ(*I*)
Graphite monochromator	*R*_int_ = 0.025
phi and ω scans	θ_max_ = 26.0°, θ_min_ = 1.5°
Absorption correction: multi-scan (*SADABS*; Sheldrick, 1996)	*h* = −17→17
*T*_min_ = 0.611, *T*_max_ = 0.624	*k* = −18→18
42971 measured reflections	*l* = −18→18

### Refinement

**Table:**

Refinement on *F*^2^	Primary atom site location: structure-invariant direct methods
Least-squares matrix: full	Secondary atom site location: difference Fourier map
*R*[*F*^2^ > 2σ(*F*^2^)] = 0.022	Hydrogen site location: inferred from neighbouring sites
*wR*(*F*^2^) = 0.058	H-atom parameters constrained
*S* = 1.13	*w* = 1/[σ^2^(*F*_o_^2^) + (0.0235*P*)^2^ + 2.1975*P*] where *P* = (*F*_o_^2^ + 2*F*_c_^2^)/3
11020 reflections	(Δ/σ)_max_ = 0.004
848 parameters	Δρ_max_ = 0.66 e Å^−^^3^
24 restraints	Δρ_min_ = −0.67 e Å^−^^3^

### Special details

**Table:**

Geometry. Bond distances, angles etc. have been calculated using the rounded fractional coordinates. All su's are estimated from the variances of the (full) variance-covariance matrix. The cell esds are taken into account in the estimation of distances, angles and torsion angles
Refinement. Refinement of F^2^ against ALL reflections. The weighted R-factor wR and goodness of fit S are based on F^2^, conventional R-factors R are based on F, with F set to zero for negative F^2^. The threshold expression of F^2^ > 2sigma(F^2^) is used only for calculating R-factors(gt) etc. and is not relevant to the choice of reflections for refinement. R-factors based on F^2^ are statistically about twice as large as those based on F, and R- factors based on ALL data will be even larger.

### Fractional atomic coordinates and isotropic or equivalent isotropic displacement parameters (Å^2^)

**Table:**

	*x*	*y*	*z*	*U*_iso_*/*U*_eq_	Occ. (<1)
Dy1	0.28833 (1)	0.97110 (1)	0.24389 (1)	0.0340 (1)	
Dy2	0.08445 (1)	0.64772 (1)	0.06388 (1)	0.0293 (1)	
O1	0.40358 (19)	1.09572 (16)	0.2241 (2)	0.0577 (9)	
O2	0.47835 (18)	1.01655 (16)	0.32745 (18)	0.0527 (8)	
O3	0.9290 (2)	1.0903 (3)	0.3411 (3)	0.0988 (15)	
O4	0.8535 (3)	1.0435 (3)	0.4309 (3)	0.0911 (16)	
O5	0.12405 (17)	0.91358 (14)	0.23748 (18)	0.0488 (7)	
O6	0.04254 (16)	0.78523 (13)	0.11067 (16)	0.0395 (7)	
O7	−0.3214 (3)	0.5943 (3)	0.1974 (3)	0.1315 (19)	
O8	−0.2587 (2)	0.5695 (2)	0.0899 (3)	0.0816 (11)	
O9	0.31791 (19)	0.85744 (14)	0.32943 (17)	0.0510 (8)	
O10	0.2210 (13)	0.7314 (10)	0.2201 (14)	0.052 (3)	0.552 (6)
O11	0.2006 (12)	0.4349 (6)	0.3129 (7)	0.108 (4)	0.552 (6)
O12	0.3409 (4)	0.4208 (3)	0.4299 (4)	0.138 (2)	
O13	0.32544 (18)	0.87284 (14)	0.13393 (18)	0.0486 (7)	
O14	0.2068 (17)	0.7471 (12)	0.0279 (17)	0.042 (3)	0.552 (6)
O15	0.7221 (3)	0.8169 (3)	0.0730 (4)	0.121 (2)	
O16	0.6931 (6)	0.9069 (6)	0.1817 (8)	0.118 (3)	0.552 (6)
O17	−0.02219 (19)	0.43184 (17)	−0.15496 (19)	0.0551 (8)	
O18	0.0549 (2)	0.58560 (17)	−0.10098 (17)	0.0579 (8)	
O19	−0.093 (5)	0.274 (3)	−0.499 (4)	0.124 (9)	0.552 (6)
O20	0.002 (3)	0.310 (3)	−0.564 (3)	0.131 (10)	0.552 (6)
O21	0.1757 (2)	0.53620 (17)	0.0789 (2)	0.0587 (9)	
O22	0.09576 (17)	0.38329 (17)	0.02968 (18)	0.0543 (8)	
O23	0.5378 (9)	0.6545 (7)	0.2803 (8)	0.097 (3)	0.552 (6)
O24	0.6252 (6)	0.5694 (7)	0.3608 (7)	0.096 (2)	0.552 (6)
N1	0.3146 (3)	1.06292 (18)	0.4102 (2)	0.0534 (10)	
N2	0.2726 (3)	1.13707 (19)	0.4196 (3)	0.0622 (11)	
N3	0.2018 (2)	1.10703 (16)	0.2480 (2)	0.0447 (9)	
N4	0.1392 (2)	1.06919 (19)	0.0772 (2)	0.0512 (9)	
N5	0.1847 (2)	0.9962 (2)	0.0805 (2)	0.0485 (9)	
N6	0.8551 (3)	1.0784 (3)	0.3639 (3)	0.0704 (14)	
N7	−0.2562 (2)	0.6148 (2)	0.1655 (3)	0.0654 (13)	
N8	0.2854 (5)	0.4694 (3)	0.3926 (5)	0.101 (2)	
N9	0.6611 (3)	0.8432 (3)	0.1008 (4)	0.110 (2)	
N10	−0.030 (5)	0.329 (5)	−0.505 (2)	0.087 (9)	0.552 (6)
N11	0.5474 (8)	0.5820 (7)	0.2990 (9)	0.065 (3)	0.552 (6)
C1	0.4830 (3)	1.0713 (2)	0.2735 (2)	0.0456 (10)	
C2	0.5837 (3)	1.1049 (2)	0.2650 (3)	0.0485 (10)	
C3	0.6724 (3)	1.0806 (2)	0.3206 (3)	0.0491 (11)	
C4	0.7627 (3)	1.1081 (2)	0.3079 (3)	0.0557 (11)	
C5	0.7681 (4)	1.1602 (3)	0.2423 (3)	0.0727 (17)	
C6	0.6801 (4)	1.1851 (4)	0.1880 (4)	0.0850 (17)	
C7	0.5876 (3)	1.1573 (3)	0.1984 (3)	0.0684 (14)	
C8	0.0542 (2)	0.83835 (19)	0.1921 (2)	0.0368 (9)	
C9	−0.0221 (2)	0.8101 (2)	0.2355 (2)	0.0398 (9)	
C10	−0.1026 (2)	0.7295 (2)	0.1832 (2)	0.0413 (10)	
C11	−0.1725 (3)	0.7025 (2)	0.2226 (3)	0.0513 (11)	
C12	−0.1656 (4)	0.7535 (3)	0.3120 (3)	0.0702 (17)	
C13	−0.0863 (4)	0.8335 (3)	0.3631 (3)	0.0757 (17)	
C14	−0.0138 (3)	0.8627 (3)	0.3259 (3)	0.0596 (14)	
C15	0.2815 (2)	0.7701 (2)	0.3055 (2)	0.0427 (9)	
C16	0.3276 (2)	0.7174 (2)	0.3800 (2)	0.0411 (9)	
C17	0.2849 (3)	0.6216 (2)	0.3564 (3)	0.0490 (11)	
C18	0.3326 (4)	0.5723 (2)	0.4209 (3)	0.0654 (13)	
C19	0.4211 (5)	0.6131 (3)	0.5060 (4)	0.091 (2)	
C20	0.4609 (4)	0.7092 (4)	0.5317 (3)	0.0933 (19)	
C21	0.4127 (3)	0.7618 (3)	0.4685 (3)	0.0651 (13)	
C22	0.3011 (2)	0.7914 (2)	0.0792 (2)	0.0404 (10)	
C23	0.3859 (2)	0.7554 (2)	0.0603 (2)	0.0392 (9)	
C24	0.4835 (3)	0.8154 (2)	0.0919 (3)	0.0534 (11)	
C25	0.5576 (3)	0.7806 (3)	0.0687 (3)	0.0627 (14)	
C26	0.5394 (3)	0.6899 (3)	0.0168 (3)	0.0631 (14)	
C27	0.4431 (3)	0.6301 (3)	−0.0120 (3)	0.0621 (13)	
C28	0.3669 (3)	0.6623 (2)	0.0094 (3)	0.0500 (11)	
C29	0.0260 (2)	0.5067 (2)	−0.1618 (2)	0.0413 (10)	
C30	0.0528 (2)	0.5006 (2)	−0.2499 (2)	0.0413 (10)	
C31	0.0034 (3)	0.4220 (2)	−0.3311 (3)	0.0508 (11)	
C32	0.0345 (4)	0.4164 (3)	−0.4083 (3)	0.0675 (16)	
C33	0.1132 (4)	0.4829 (4)	−0.4071 (4)	0.084 (2)	
C34	0.1607 (4)	0.5611 (4)	−0.3273 (4)	0.086 (2)	
C35	0.1301 (3)	0.5707 (3)	−0.2492 (3)	0.0609 (12)	
C36	0.1746 (2)	0.4516 (2)	0.0751 (2)	0.0399 (9)	
C37	0.2766 (2)	0.4314 (2)	0.1309 (2)	0.0368 (9)	
C38	0.3648 (3)	0.5048 (2)	0.1863 (2)	0.0483 (10)	
C39	0.4567 (3)	0.4834 (4)	0.2389 (3)	0.0678 (16)	
C40	0.4638 (4)	0.3935 (5)	0.2368 (4)	0.086 (2)	
C41	0.3769 (4)	0.3214 (4)	0.1822 (4)	0.0787 (17)	
C42	0.2830 (3)	0.3397 (2)	0.1286 (3)	0.0535 (11)	
C43	0.3712 (4)	1.0616 (3)	0.5023 (3)	0.0725 (14)	
C44	0.3677 (6)	1.1353 (4)	0.5717 (4)	0.105 (2)	
C45	0.3065 (5)	1.1821 (3)	0.5178 (4)	0.097 (2)	
C46	0.2056 (3)	1.1571 (2)	0.3338 (3)	0.0560 (13)	
C47	0.1488 (4)	1.2217 (3)	0.3384 (5)	0.084 (2)	
C48	0.0890 (4)	1.2382 (3)	0.2532 (6)	0.096 (2)	
C49	0.0825 (3)	1.1894 (3)	0.1616 (4)	0.0776 (16)	
C50	0.1413 (3)	1.1238 (2)	0.1650 (3)	0.0510 (13)	
C51	0.0960 (3)	1.0761 (3)	−0.0173 (3)	0.0741 (16)	
C52	0.1132 (3)	1.0081 (3)	−0.0761 (3)	0.0746 (16)	
C53	0.1689 (3)	0.9601 (3)	−0.0123 (3)	0.0605 (12)	
O20'	0.038 (4)	0.335 (4)	−0.545 (3)	0.131 (10)	0.448 (6)
O23'	0.5492 (14)	0.6242 (11)	0.3178 (12)	0.097 (3)	0.448 (6)
O10'	0.1998 (17)	0.7139 (14)	0.2273 (18)	0.052 (3)	0.448 (6)
O11'	0.1934 (14)	0.4500 (8)	0.3720 (15)	0.126 (7)	0.448 (6)
O14'	0.214 (2)	0.7289 (15)	0.042 (2)	0.042 (3)	0.448 (6)
O16'	0.6648 (8)	0.9344 (7)	0.1136 (10)	0.118 (3)	0.448 (6)
O19'	−0.108 (7)	0.296 (3)	−0.508 (5)	0.124 (9)	0.448 (6)
N10'	−0.007 (6)	0.337 (6)	−0.483 (3)	0.087 (9)	0.448 (6)
N11'	0.5531 (11)	0.5397 (8)	0.3049 (11)	0.065 (3)	0.448 (6)
O24'	0.6316 (8)	0.5128 (8)	0.3434 (9)	0.096 (2)	0.448 (6)
H3	0.67110	1.04600	0.36610	0.0590*	
H5	0.83000	1.17830	0.23490	0.0870*	
H24	0.49880	0.87770	0.12780	0.0640*	
H26	0.59090	0.66910	0.00150	0.0750*	
H27	0.42910	0.56750	−0.04620	0.0740*	
H28	0.30180	0.62110	−0.01060	0.0600*	
H31	−0.04940	0.37430	−0.33330	0.0610*	
H33	0.13420	0.47550	−0.45920	0.1010*	
H34	0.21380	0.60800	−0.32560	0.1040*	
H35	0.16180	0.62460	−0.19590	0.0730*	
H38	0.36230	0.56690	0.18820	0.0580*	
H40	0.52730	0.38150	0.27230	0.1040*	
H41	0.38060	0.25960	0.18070	0.0940*	
H42	0.22380	0.28990	0.09100	0.0640*	
H43	0.40860	1.01710	0.51870	0.0870*	
H44	0.40090	1.14910	0.64090	0.1260*	
H45	0.29000	1.23600	0.54300	0.1160*	
H47	0.15160	1.25350	0.39920	0.1010*	
H48	0.05110	1.28300	0.25520	0.1150*	
H49	0.04120	1.20010	0.10180	0.0930*	
H51	0.06030	1.12050	−0.03800	0.0890*	
H52	0.09250	0.99570	−0.14480	0.0890*	
H53	0.19210	0.90890	−0.03280	0.0730*	
H6	0.68240	1.22100	0.14370	0.1020*	
H7	0.52810	1.17390	0.16060	0.0820*	
H10	−0.10950	0.69400	0.12220	0.0500*	
H12	−0.21400	0.73400	0.33710	0.0850*	
H13	−0.08070	0.86890	0.42360	0.0910*	
H14	0.04010	0.91730	0.36140	0.0720*	
H17	0.22510	0.59130	0.29830	0.0590*	
H19	0.45400	0.57680	0.54590	0.1100*	
H20	0.51960	0.73910	0.59090	0.1110*	
H21	0.43840	0.82700	0.48640	0.0780*	

### Atomic displacement parameters (Å^2^)

**Table:**

	*U*^11^	*U*^22^	*U*^33^	*U*^12^	*U*^13^	*U*^23^
Dy1	0.0390 (1)	0.0263 (1)	0.0321 (1)	0.0088 (1)	0.0106 (1)	0.0058 (1)
Dy2	0.0289 (1)	0.0241 (1)	0.0308 (1)	0.0085 (1)	0.0087 (1)	0.0040 (1)
O1	0.0515 (14)	0.0458 (13)	0.0725 (18)	0.0122 (11)	0.0169 (13)	0.0270 (12)
O2	0.0466 (13)	0.0539 (13)	0.0456 (14)	0.0027 (10)	0.0076 (11)	0.0193 (11)
O3	0.0574 (19)	0.103 (3)	0.130 (3)	0.0175 (17)	0.041 (2)	0.015 (2)
O4	0.074 (2)	0.104 (3)	0.089 (3)	0.0355 (19)	0.0183 (19)	0.033 (2)
O5	0.0477 (13)	0.0326 (10)	0.0584 (15)	0.0039 (9)	0.0203 (12)	0.0038 (10)
O6	0.0451 (12)	0.0308 (9)	0.0488 (13)	0.0148 (9)	0.0252 (10)	0.0081 (9)
O7	0.107 (3)	0.140 (3)	0.144 (4)	−0.031 (2)	0.093 (3)	−0.001 (3)
O8	0.076 (2)	0.0680 (18)	0.089 (2)	−0.0116 (15)	0.0469 (19)	−0.0062 (16)
O9	0.0629 (15)	0.0348 (11)	0.0460 (13)	0.0095 (10)	0.0117 (12)	0.0154 (10)
O10	0.050 (6)	0.032 (5)	0.044 (3)	−0.014 (4)	−0.003 (3)	0.014 (3)
O11	0.151 (9)	0.039 (3)	0.101 (7)	−0.004 (4)	0.031 (6)	0.016 (4)
O12	0.202 (5)	0.060 (2)	0.152 (4)	0.054 (3)	0.053 (4)	0.051 (2)
O13	0.0517 (13)	0.0397 (11)	0.0555 (14)	0.0112 (10)	0.0279 (12)	0.0031 (10)
O14	0.043 (3)	0.029 (6)	0.061 (6)	0.011 (4)	0.026 (4)	0.019 (3)
O15	0.061 (2)	0.099 (3)	0.204 (5)	0.0131 (18)	0.072 (3)	0.016 (3)
O16	0.067 (4)	0.085 (4)	0.165 (7)	−0.007 (3)	0.049 (5)	−0.029 (4)
O17	0.0608 (15)	0.0584 (14)	0.0568 (15)	0.0169 (12)	0.0302 (13)	0.0261 (12)
O18	0.0690 (16)	0.0609 (15)	0.0369 (13)	0.0142 (12)	0.0218 (12)	−0.0002 (11)
O19	0.172 (18)	0.076 (16)	0.078 (9)	0.023 (12)	0.026 (10)	−0.021 (11)
O20	0.16 (2)	0.151 (19)	0.059 (12)	0.058 (15)	0.041 (13)	−0.035 (8)
O21	0.0644 (16)	0.0552 (14)	0.0718 (17)	0.0395 (12)	0.0304 (14)	0.0254 (12)
O22	0.0328 (12)	0.0683 (15)	0.0464 (14)	0.0111 (11)	0.0068 (10)	0.0016 (12)
O23	0.074 (3)	0.081 (6)	0.097 (7)	−0.011 (4)	0.026 (5)	−0.019 (4)
O24	0.043 (2)	0.137 (6)	0.073 (4)	0.003 (4)	0.000 (2)	0.017 (5)
N1	0.075 (2)	0.0376 (14)	0.0430 (17)	0.0071 (13)	0.0259 (15)	0.0038 (12)
N2	0.092 (2)	0.0386 (15)	0.061 (2)	0.0106 (15)	0.045 (2)	0.0016 (14)
N3	0.0488 (15)	0.0282 (12)	0.0622 (18)	0.0115 (11)	0.0266 (14)	0.0144 (12)
N4	0.0386 (14)	0.0497 (15)	0.0631 (19)	0.0124 (12)	0.0111 (14)	0.0306 (14)
N5	0.0460 (15)	0.0551 (16)	0.0456 (17)	0.0200 (13)	0.0141 (13)	0.0202 (13)
N6	0.051 (2)	0.066 (2)	0.077 (3)	0.0081 (16)	0.0181 (19)	0.0032 (19)
N7	0.0507 (18)	0.067 (2)	0.083 (3)	0.0026 (15)	0.0379 (19)	0.0202 (18)
N8	0.146 (5)	0.047 (2)	0.124 (5)	0.027 (3)	0.059 (4)	0.045 (3)
N9	0.052 (2)	0.078 (3)	0.177 (5)	−0.0016 (19)	0.057 (3)	−0.023 (3)
N10	0.12 (2)	0.097 (10)	0.022 (13)	0.054 (15)	0.007 (13)	−0.009 (13)
N11	0.041 (2)	0.084 (7)	0.052 (3)	0.004 (5)	0.012 (2)	0.006 (5)
C1	0.0457 (18)	0.0337 (15)	0.0420 (18)	−0.0002 (13)	0.0094 (15)	0.0029 (13)
C2	0.0490 (19)	0.0403 (16)	0.0444 (19)	0.0012 (14)	0.0123 (16)	0.0082 (14)
C3	0.052 (2)	0.0444 (17)	0.0407 (18)	0.0045 (15)	0.0127 (16)	0.0089 (14)
C4	0.049 (2)	0.0493 (19)	0.054 (2)	0.0027 (15)	0.0149 (17)	0.0020 (16)
C5	0.063 (3)	0.077 (3)	0.074 (3)	−0.001 (2)	0.032 (2)	0.020 (2)
C6	0.080 (3)	0.096 (3)	0.082 (3)	0.004 (3)	0.033 (3)	0.049 (3)
C7	0.062 (2)	0.069 (2)	0.068 (3)	0.007 (2)	0.017 (2)	0.034 (2)
C8	0.0401 (16)	0.0288 (13)	0.0453 (18)	0.0153 (12)	0.0187 (14)	0.0104 (12)
C9	0.0440 (17)	0.0371 (15)	0.0420 (17)	0.0160 (13)	0.0202 (14)	0.0086 (13)
C10	0.0434 (17)	0.0414 (15)	0.0448 (18)	0.0148 (13)	0.0234 (15)	0.0099 (13)
C11	0.051 (2)	0.0530 (19)	0.059 (2)	0.0158 (16)	0.0315 (18)	0.0151 (16)
C12	0.077 (3)	0.076 (3)	0.075 (3)	0.020 (2)	0.052 (3)	0.016 (2)
C13	0.098 (3)	0.082 (3)	0.059 (3)	0.026 (3)	0.051 (3)	0.004 (2)
C14	0.073 (3)	0.053 (2)	0.051 (2)	0.0110 (18)	0.032 (2)	0.0007 (16)
C15	0.0442 (17)	0.0385 (15)	0.0344 (17)	0.0085 (13)	0.0055 (14)	0.0093 (13)
C16	0.0493 (18)	0.0332 (14)	0.0324 (16)	0.0070 (13)	0.0096 (14)	0.0082 (12)
C17	0.058 (2)	0.0356 (15)	0.048 (2)	0.0082 (14)	0.0183 (17)	0.0098 (14)
C18	0.101 (3)	0.0413 (18)	0.057 (2)	0.021 (2)	0.031 (2)	0.0216 (17)
C19	0.136 (5)	0.069 (3)	0.063 (3)	0.044 (3)	0.018 (3)	0.040 (2)
C20	0.103 (4)	0.084 (3)	0.052 (3)	0.015 (3)	−0.010 (3)	0.022 (2)
C21	0.075 (3)	0.0438 (18)	0.044 (2)	0.0058 (17)	−0.0064 (19)	0.0116 (16)
C22	0.0413 (17)	0.0393 (15)	0.0431 (18)	0.0090 (13)	0.0215 (15)	0.0093 (13)
C23	0.0398 (16)	0.0387 (15)	0.0400 (17)	0.0100 (12)	0.0181 (14)	0.0092 (13)
C24	0.0422 (18)	0.0444 (17)	0.062 (2)	0.0041 (14)	0.0205 (17)	−0.0045 (16)
C25	0.0394 (19)	0.060 (2)	0.080 (3)	0.0047 (16)	0.0264 (19)	0.0022 (19)
C26	0.052 (2)	0.062 (2)	0.080 (3)	0.0217 (18)	0.035 (2)	0.007 (2)
C27	0.062 (2)	0.0450 (18)	0.080 (3)	0.0138 (17)	0.037 (2)	0.0017 (18)
C28	0.0507 (19)	0.0395 (16)	0.060 (2)	0.0075 (14)	0.0283 (18)	0.0056 (15)
C29	0.0412 (16)	0.0485 (17)	0.0344 (17)	0.0147 (14)	0.0146 (14)	0.0112 (14)
C30	0.0480 (18)	0.0410 (15)	0.0360 (17)	0.0133 (13)	0.0177 (14)	0.0106 (13)
C31	0.063 (2)	0.0459 (17)	0.0428 (19)	0.0204 (16)	0.0194 (17)	0.0096 (15)
C32	0.093 (3)	0.075 (3)	0.043 (2)	0.044 (2)	0.030 (2)	0.0107 (19)
C33	0.101 (4)	0.120 (4)	0.065 (3)	0.053 (3)	0.057 (3)	0.034 (3)
C34	0.084 (3)	0.103 (4)	0.090 (4)	0.012 (3)	0.056 (3)	0.036 (3)
C35	0.064 (2)	0.061 (2)	0.054 (2)	0.0015 (18)	0.027 (2)	0.0146 (18)
C36	0.0382 (16)	0.0457 (16)	0.0357 (16)	0.0210 (14)	0.0124 (14)	0.0075 (13)
C37	0.0334 (15)	0.0376 (14)	0.0383 (16)	0.0134 (12)	0.0131 (13)	0.0075 (12)
C38	0.0411 (17)	0.0530 (18)	0.0443 (19)	0.0070 (14)	0.0175 (15)	0.0026 (15)
C39	0.0320 (18)	0.119 (4)	0.037 (2)	0.010 (2)	0.0090 (15)	0.005 (2)
C40	0.061 (3)	0.157 (5)	0.062 (3)	0.067 (3)	0.024 (2)	0.047 (3)
C41	0.092 (3)	0.090 (3)	0.093 (3)	0.066 (3)	0.049 (3)	0.053 (3)
C42	0.058 (2)	0.0457 (18)	0.068 (2)	0.0253 (16)	0.0300 (19)	0.0217 (17)
C43	0.102 (3)	0.061 (2)	0.041 (2)	0.003 (2)	0.025 (2)	0.0086 (18)
C44	0.172 (6)	0.076 (3)	0.051 (3)	0.001 (4)	0.052 (4)	−0.003 (2)
C45	0.160 (5)	0.062 (3)	0.072 (3)	0.012 (3)	0.073 (4)	−0.011 (2)
C46	0.066 (2)	0.0283 (15)	0.083 (3)	0.0068 (15)	0.047 (2)	0.0066 (16)
C47	0.090 (3)	0.044 (2)	0.134 (5)	0.025 (2)	0.068 (4)	0.006 (2)
C48	0.073 (3)	0.042 (2)	0.181 (6)	0.027 (2)	0.063 (4)	0.017 (3)
C49	0.049 (2)	0.048 (2)	0.137 (4)	0.0214 (18)	0.029 (3)	0.040 (3)
C50	0.0394 (17)	0.0357 (15)	0.080 (3)	0.0110 (13)	0.0233 (18)	0.0224 (17)
C51	0.060 (2)	0.073 (3)	0.070 (3)	0.010 (2)	−0.002 (2)	0.043 (2)
C52	0.064 (3)	0.089 (3)	0.050 (2)	0.001 (2)	0.002 (2)	0.034 (2)
C53	0.056 (2)	0.075 (2)	0.041 (2)	0.0142 (19)	0.0110 (17)	0.0153 (18)
O20'	0.16 (2)	0.151 (19)	0.059 (12)	0.058 (15)	0.041 (13)	−0.035 (8)
O23'	0.074 (3)	0.081 (6)	0.097 (7)	−0.011 (4)	0.026 (5)	−0.019 (4)
O10'	0.050 (6)	0.032 (5)	0.044 (3)	−0.014 (4)	−0.003 (3)	0.014 (3)
O11'	0.127 (9)	0.051 (5)	0.213 (17)	0.008 (5)	0.094 (12)	0.026 (8)
O14'	0.043 (3)	0.029 (6)	0.061 (6)	0.011 (4)	0.026 (4)	0.019 (3)
O16'	0.067 (4)	0.085 (4)	0.165 (7)	−0.007 (3)	0.049 (5)	−0.029 (4)
O19'	0.172 (18)	0.076 (16)	0.078 (9)	0.023 (12)	0.026 (10)	−0.021 (11)
N10'	0.12 (2)	0.097 (10)	0.022 (13)	0.054 (15)	0.007 (13)	−0.009 (13)
N11'	0.041 (2)	0.084 (7)	0.052 (3)	0.004 (5)	0.012 (2)	0.006 (5)
O24'	0.043 (2)	0.137 (6)	0.073 (4)	0.003 (4)	0.000 (2)	0.017 (5)

### Geometric parameters (Å, º)

**Table:**

Dy1—O1	2.404 (3)	C9—C10	1.380 (4)
Dy1—O2	2.433 (3)	C9—C14	1.387 (5)
Dy1—O5	2.314 (3)	C10—C11	1.374 (5)
Dy1—O9	2.307 (2)	C11—C12	1.374 (6)
Dy1—O13	2.274 (3)	C12—C13	1.365 (7)
Dy1—N1	2.472 (3)	C13—C14	1.389 (7)
Dy1—N3	2.549 (3)	C15—C16	1.497 (4)
Dy1—N5	2.478 (3)	C16—C17	1.380 (4)
Dy2—O6	2.294 (2)	C16—C21	1.373 (5)
Dy2—O10	2.358 (19)	C17—C18	1.369 (5)
Dy2—O14	2.38 (2)	C18—C19	1.361 (8)
Dy2—O18	2.322 (2)	C19—C20	1.375 (8)
Dy2—O21	2.272 (3)	C20—C21	1.397 (7)
Dy2—O10'	2.29 (2)	C22—C23	1.499 (4)
Dy2—O14'	2.20 (3)	C23—C24	1.378 (5)
Dy2—O17^i^	2.277 (3)	C23—C28	1.386 (5)
Dy2—O22^i^	2.339 (3)	C24—C25	1.377 (6)
O1—C1	1.256 (5)	C25—C26	1.362 (6)
O2—C1	1.259 (4)	C26—C27	1.370 (6)
O3—N6	1.218 (6)	C27—C28	1.375 (6)
O4—N6	1.216 (6)	C29—C30	1.494 (4)
O5—C8	1.249 (4)	C30—C31	1.385 (5)
O6—C8	1.263 (4)	C30—C35	1.381 (6)
O7—N7	1.213 (6)	C31—C32	1.376 (7)
O8—N7	1.193 (5)	C32—C33	1.359 (8)
O9—C15	1.250 (4)	C33—C34	1.370 (8)
O10—C15	1.207 (19)	C34—C35	1.384 (7)
O10'—C15	1.32 (2)	C36—C37	1.497 (4)
O11—N8	1.283 (14)	C37—C42	1.382 (4)
O11'—N8	1.20 (2)	C37—C38	1.379 (5)
O12—N8	1.215 (8)	C38—C39	1.380 (6)
O13—C22	1.247 (4)	C39—C40	1.358 (9)
O14—C22	1.26 (2)	C40—C41	1.357 (9)
O14'—C22	1.28 (3)	C41—C42	1.384 (7)
O15—N9	1.186 (7)	C43—C44	1.391 (8)
O16—N9	1.267 (12)	C44—C45	1.337 (9)
O16'—N9	1.329 (12)	C46—C47	1.368 (7)
O17—C29	1.251 (4)	C47—C48	1.338 (10)
O18—C29	1.247 (4)	C48—C49	1.392 (9)
O19—N10	1.15 (10)	C49—C50	1.388 (6)
O19'—N10'	1.34 (13)	C51—C52	1.336 (6)
O20—N10	1.15 (7)	C52—C53	1.390 (6)
O20'—N10'	1.31 (9)	C3—H3	0.9300
O21—C36	1.251 (4)	C5—H5	0.9300
O22—C36	1.249 (4)	C6—H6	0.9300
O23—N11	1.183 (15)	C7—H7	0.9300
O23'—N11'	1.26 (2)	C10—H10	0.9300
O24—N11	1.222 (15)	C12—H12	0.9300
O24'—N11'	1.23 (2)	C13—H13	0.9300
N1—C43	1.320 (5)	C14—H14	0.9300
N1—N2	1.354 (5)	C17—H17	0.9300
N2—C45	1.360 (7)	C19—H19	0.9300
N2—C46	1.406 (6)	C20—H20	0.9300
N3—C46	1.340 (5)	C21—H21	0.9300
N3—C50	1.319 (5)	C24—H24	0.9300
N4—C51	1.348 (5)	C26—H26	0.9300
N4—N5	1.358 (4)	C27—H27	0.9300
N4—C50	1.402 (5)	C28—H28	0.9300
N5—C53	1.313 (5)	C31—H31	0.9300
N6—C4	1.465 (6)	C33—H33	0.9300
N7—C11	1.476 (5)	C34—H34	0.9300
N8—C18	1.482 (6)	C35—H35	0.9300
N9—C25	1.459 (7)	C38—H38	0.9300
N10—C32	1.60 (5)	C40—H40	0.9300
N10'—C32	1.34 (6)	C41—H41	0.9300
N11—C39	1.632 (13)	C42—H42	0.9300
N11'—C39	1.369 (16)	C43—H43	0.9300
C1—C2	1.498 (6)	C44—H44	0.9300
C2—C3	1.381 (6)	C45—H45	0.9300
C2—C7	1.385 (6)	C47—H47	0.9300
C3—C4	1.376 (6)	C48—H48	0.9300
C4—C5	1.374 (6)	C49—H49	0.9300
C5—C6	1.372 (8)	C51—H51	0.9300
C6—C7	1.388 (8)	C52—H52	0.9300
C8—C9	1.500 (4)	C53—H53	0.9300
			
O1—Dy1—O2	53.79 (9)	C9—C14—C13	119.7 (4)
O1—Dy1—O5	146.83 (9)	O9—C15—O10	118.4 (8)
O1—Dy1—O9	132.71 (10)	O9—C15—C16	117.9 (3)
O1—Dy1—O13	85.78 (9)	O9—C15—O10'	130.9 (10)
O1—Dy1—N1	92.97 (10)	O10—C15—C16	123.0 (8)
O1—Dy1—N3	76.10 (9)	O10'—C15—C16	110.9 (10)
O1—Dy1—N5	75.17 (10)	C15—C16—C21	121.4 (3)
O2—Dy1—O5	152.54 (8)	C17—C16—C21	120.1 (3)
O2—Dy1—O9	79.08 (9)	C15—C16—C17	118.5 (3)
O2—Dy1—O13	80.33 (9)	C16—C17—C18	118.3 (4)
O2—Dy1—N1	79.47 (12)	N8—C18—C19	120.1 (4)
O2—Dy1—N3	115.08 (9)	C17—C18—C19	123.0 (4)
O2—Dy1—N5	124.91 (9)	N8—C18—C17	116.9 (4)
O5—Dy1—O9	78.83 (9)	C18—C19—C20	118.6 (5)
O5—Dy1—O13	112.50 (9)	C19—C20—C21	119.7 (4)
O5—Dy1—N1	81.09 (11)	C16—C21—C20	120.1 (4)
O5—Dy1—N3	72.07 (8)	O14'—C22—C23	113.0 (13)
O5—Dy1—N5	82.41 (9)	O14—C22—C23	120.0 (11)
O9—Dy1—O13	82.31 (8)	O13—C22—O14	121.1 (11)
O9—Dy1—N1	80.84 (9)	O13—C22—C23	117.7 (3)
O9—Dy1—N3	136.38 (9)	O13—C22—O14'	129.0 (13)
O9—Dy1—N5	143.44 (9)	C22—C23—C28	120.7 (3)
O13—Dy1—N1	155.77 (11)	C24—C23—C28	119.2 (3)
O13—Dy1—N3	138.76 (8)	C22—C23—C24	120.1 (3)
O13—Dy1—N5	76.31 (9)	C23—C24—C25	118.2 (3)
N1—Dy1—N3	63.33 (10)	N9—C25—C24	119.0 (4)
N1—Dy1—N5	126.76 (10)	N9—C25—C26	117.7 (4)
N3—Dy1—N5	63.43 (9)	C24—C25—C26	123.3 (4)
O6—Dy2—O10	74.2 (4)	C25—C26—C27	118.1 (4)
O6—Dy2—O14	83.5 (5)	C26—C27—C28	120.3 (4)
O6—Dy2—O18	120.81 (8)	C23—C28—C27	120.9 (4)
O6—Dy2—O21	157.47 (9)	O17—C29—O18	125.8 (3)
O6—Dy2—O10'	74.9 (6)	O17—C29—C30	117.0 (3)
O6—Dy2—O14'	89.4 (7)	O18—C29—C30	117.1 (3)
O6—Dy2—O17^i^	96.50 (9)	C29—C30—C31	119.9 (3)
O6—Dy2—O22^i^	75.08 (9)	C29—C30—C35	120.5 (3)
O10—Dy2—O14	74.4 (7)	C31—C30—C35	119.5 (3)
O10—Dy2—O18	141.0 (5)	C30—C31—C32	118.2 (4)
O10—Dy2—O21	83.4 (4)	N10—C32—C33	120 (2)
O10—Dy2—O17^i^	84.3 (4)	C31—C32—C33	123.1 (4)
O10—Dy2—O22^i^	143.1 (5)	N10'—C32—C31	118 (4)
O14—Dy2—O18	72.3 (5)	N10'—C32—C33	118 (4)
O14—Dy2—O21	92.8 (5)	N10—C32—C31	117 (2)
O14—Dy2—O17^i^	157.9 (6)	C32—C33—C34	118.4 (5)
O14—Dy2—O22^i^	121.6 (6)	C33—C34—C35	120.4 (5)
O18—Dy2—O21	78.58 (10)	C30—C35—C34	120.3 (4)
O10'—Dy2—O18	149.3 (7)	O21—C36—C37	116.7 (3)
O14'—Dy2—O18	74.0 (7)	O21—C36—O22	125.6 (3)
O17^i^—Dy2—O18	124.71 (9)	O22—C36—C37	117.7 (3)
O18—Dy2—O22^i^	73.73 (10)	C38—C37—C42	119.6 (3)
O10'—Dy2—O21	82.6 (6)	C36—C37—C38	119.9 (3)
O14'—Dy2—O21	85.2 (7)	C36—C37—C42	120.6 (3)
O17^i^—Dy2—O21	78.66 (10)	C37—C38—C39	118.1 (3)
O21—Dy2—O22^i^	124.61 (9)	N11—C39—C40	129.1 (6)
O10'—Dy2—O14'	80.4 (9)	C38—C39—C40	122.7 (5)
O10'—Dy2—O17^i^	74.1 (6)	N11'—C39—C38	131.6 (7)
O10'—Dy2—O22^i^	136.8 (7)	N11'—C39—C40	105.6 (7)
O14'—Dy2—O17^i^	151.3 (7)	N11—C39—C38	108.2 (6)
O14'—Dy2—O22^i^	129.3 (7)	C39—C40—C41	119.0 (6)
O17^i^—Dy2—O22^i^	79.31 (9)	C40—C41—C42	120.2 (5)
Dy1—O1—C1	92.2 (2)	C37—C42—C41	120.4 (4)
Dy1—O2—C1	90.7 (2)	N1—C43—C44	111.2 (5)
Dy1—O5—C8	134.5 (2)	C43—C44—C45	105.4 (5)
Dy2—O6—C8	135.76 (19)	N2—C45—C44	108.0 (5)
Dy1—O9—C15	133.2 (2)	N2—C46—N3	114.3 (3)
Dy2—O10—C15	171.1 (14)	N2—C46—C47	122.4 (4)
Dy2—O10'—C15	159.6 (16)	N3—C46—C47	123.4 (4)
Dy1—O13—C22	145.6 (2)	C46—C47—C48	118.4 (6)
Dy2—O14—C22	132.3 (14)	C47—C48—C49	121.1 (5)
Dy2—O14'—C22	148.8 (19)	C48—C49—C50	115.9 (5)
Dy2^i^—O17—C29	149.3 (2)	N3—C50—C49	124.2 (4)
Dy2—O18—C29	138.4 (2)	N4—C50—C49	120.9 (4)
Dy2—O21—C36	147.8 (2)	N3—C50—N4	114.9 (3)
Dy2^i^—O22—C36	139.9 (2)	N4—C51—C52	108.2 (4)
Dy1—N1—N2	120.7 (2)	C51—C52—C53	105.0 (4)
Dy1—N1—C43	133.6 (3)	N5—C53—C52	111.6 (4)
N2—N1—C43	105.4 (3)	C2—C3—H3	120.00
N1—N2—C45	109.9 (4)	C4—C3—H3	120.00
C45—N2—C46	130.3 (4)	C4—C5—H5	121.00
N1—N2—C46	119.8 (3)	C6—C5—H5	121.00
Dy1—N3—C50	121.3 (2)	C5—C6—H6	120.00
Dy1—N3—C46	121.2 (2)	C7—C6—H6	120.00
C46—N3—C50	117.0 (3)	C2—C7—H7	120.00
N5—N4—C51	110.3 (3)	C6—C7—H7	120.00
N5—N4—C50	120.0 (3)	C9—C10—H10	121.00
C50—N4—C51	129.7 (3)	C11—C10—H10	120.00
N4—N5—C53	104.9 (3)	C11—C12—H12	121.00
Dy1—N5—N4	119.76 (19)	C13—C12—H12	121.00
Dy1—N5—C53	134.9 (3)	C12—C13—H13	120.00
O4—N6—C4	117.9 (4)	C14—C13—H13	120.00
O3—N6—C4	118.3 (4)	C9—C14—H14	120.00
O3—N6—O4	123.8 (5)	C13—C14—H14	120.00
O7—N7—O8	123.3 (4)	C16—C17—H17	121.00
O7—N7—C11	117.2 (4)	C18—C17—H17	121.00
O8—N7—C11	119.5 (3)	C18—C19—H19	121.00
O11—N8—O12	122.2 (7)	C20—C19—H19	121.00
O11'—N8—O12	121.9 (9)	C19—C20—H20	120.00
O11'—N8—C18	113.0 (8)	C21—C20—H20	120.00
O11—N8—C18	117.5 (6)	C16—C21—H21	120.00
O12—N8—C18	117.5 (6)	C20—C21—H21	120.00
O15—N9—O16'	116.4 (7)	C23—C24—H24	121.00
O15—N9—O16	120.0 (6)	C25—C24—H24	121.00
O15—N9—C25	119.6 (5)	C25—C26—H26	121.00
O16'—N9—C25	115.4 (7)	C27—C26—H26	121.00
O16—N9—C25	115.8 (6)	C26—C27—H27	120.00
O19—N10—C32	116 (4)	C28—C27—H27	120.00
O20—N10—C32	121 (6)	C23—C28—H28	120.00
O19—N10—O20	121 (6)	C27—C28—H28	120.00
O19'—N10'—C32	115 (6)	C30—C31—H31	121.00
O19'—N10'—O20'	125 (5)	C32—C31—H31	121.00
O20'—N10'—C32	113 (7)	C32—C33—H33	121.00
O23—N11—C39	122.6 (11)	C34—C33—H33	121.00
O24—N11—C39	111.3 (9)	C33—C34—H34	120.00
O23—N11—O24	126.0 (12)	C35—C34—H34	120.00
O23'—N11'—C39	108.9 (14)	C30—C35—H35	120.00
O24'—N11'—C39	125.9 (12)	C34—C35—H35	120.00
O23'—N11'—O24'	125.1 (16)	C37—C38—H38	121.00
O1—C1—C2	119.5 (3)	C39—C38—H38	121.00
O2—C1—C2	119.5 (3)	C39—C40—H40	121.00
O1—C1—O2	121.0 (4)	C41—C40—H40	120.00
C1—C2—C3	120.2 (3)	C40—C41—H41	120.00
C3—C2—C7	119.4 (4)	C42—C41—H41	120.00
C1—C2—C7	120.3 (4)	C37—C42—H42	120.00
C2—C3—C4	119.3 (3)	C41—C42—H42	120.00
C3—C4—C5	122.1 (4)	N1—C43—H43	124.00
N6—C4—C3	118.9 (3)	C44—C43—H43	124.00
N6—C4—C5	119.0 (4)	C43—C44—H44	127.00
C4—C5—C6	118.4 (5)	C45—C44—H44	127.00
C5—C6—C7	120.7 (5)	N2—C45—H45	126.00
C2—C7—C6	120.1 (4)	C44—C45—H45	126.00
O6—C8—C9	117.8 (3)	C46—C47—H47	121.00
O5—C8—O6	124.0 (3)	C48—C47—H47	121.00
O5—C8—C9	118.2 (3)	C47—C48—H48	119.00
C8—C9—C14	121.9 (3)	C49—C48—H48	119.00
C10—C9—C14	119.7 (3)	C48—C49—H49	122.00
C8—C9—C10	118.5 (2)	C50—C49—H49	122.00
C9—C10—C11	119.0 (3)	N4—C51—H51	126.00
N7—C11—C12	120.3 (4)	C52—C51—H51	126.00
C10—C11—C12	122.2 (4)	C51—C52—H52	127.00
N7—C11—C10	117.4 (3)	C53—C52—H52	128.00
C11—C12—C13	118.5 (5)	N5—C53—H53	124.00
C12—C13—C14	120.9 (4)	C52—C53—H53	124.00
			
O2—Dy1—O1—C1	8.58 (18)	C45—N2—C46—N3	169.9 (5)
O5—Dy1—O1—C1	161.44 (18)	N1—N2—C45—C44	−1.9 (7)
O9—Dy1—O1—C1	3.0 (2)	N1—N2—C46—N3	−9.4 (6)
O13—Dy1—O1—C1	−72.5 (2)	Dy1—N3—C46—N2	8.7 (4)
N1—Dy1—O1—C1	83.2 (2)	Dy1—N3—C50—N4	−7.3 (4)
N3—Dy1—O1—C1	144.8 (2)	C46—N3—C50—N4	−179.5 (3)
N5—Dy1—O1—C1	−149.5 (2)	C50—N3—C46—C47	1.5 (6)
O1—Dy1—O2—C1	−8.55 (18)	Dy1—N3—C50—C49	172.3 (3)
O5—Dy1—O2—C1	−155.8 (2)	C46—N3—C50—C49	0.1 (6)
O9—Dy1—O2—C1	167.2 (2)	C50—N3—C46—N2	−179.1 (3)
O13—Dy1—O2—C1	83.29 (19)	Dy1—N3—C46—C47	−170.7 (4)
N1—Dy1—O2—C1	−110.2 (2)	C51—N4—N5—C53	0.4 (4)
N3—Dy1—O2—C1	−56.4 (2)	C51—N4—C50—C49	8.9 (6)
N5—Dy1—O2—C1	17.6 (2)	C50—N4—N5—C53	179.8 (4)
O1—Dy1—O5—C8	124.8 (3)	C51—N4—N5—Dy1	173.7 (3)
O2—Dy1—O5—C8	−108.2 (3)	C50—N4—C51—C52	−179.5 (4)
O9—Dy1—O5—C8	−71.2 (3)	N5—N4—C51—C52	−0.2 (5)
O13—Dy1—O5—C8	5.6 (3)	N5—N4—C50—C49	−170.4 (4)
N1—Dy1—O5—C8	−153.5 (3)	C50—N4—N5—Dy1	−6.9 (4)
N3—Dy1—O5—C8	141.8 (3)	C51—N4—C50—N3	−171.5 (4)
N5—Dy1—O5—C8	77.3 (3)	N5—N4—C50—N3	9.2 (5)
O1—Dy1—O9—C15	−130.3 (3)	N4—N5—C53—C52	−0.4 (5)
O2—Dy1—O9—C15	−135.0 (3)	Dy1—N5—C53—C52	−172.2 (3)
O5—Dy1—O9—C15	61.5 (3)	O3—N6—C4—C3	168.8 (4)
O13—Dy1—O9—C15	−53.4 (3)	O3—N6—C4—C5	−9.4 (6)
N1—Dy1—O9—C15	144.1 (3)	O4—N6—C4—C3	−11.5 (6)
N3—Dy1—O9—C15	110.0 (3)	O4—N6—C4—C5	170.4 (4)
N5—Dy1—O9—C15	1.0 (4)	O8—N7—C11—C12	−177.2 (4)
O1—Dy1—O13—C22	−168.1 (4)	O8—N7—C11—C10	2.2 (5)
O2—Dy1—O13—C22	137.9 (4)	O7—N7—C11—C12	5.1 (6)
O5—Dy1—O13—C22	−16.7 (4)	O7—N7—C11—C10	−175.6 (4)
O9—Dy1—O13—C22	57.7 (4)	O11—N8—C18—C17	−3.6 (12)
N1—Dy1—O13—C22	104.0 (4)	O11—N8—C18—C19	178.4 (10)
N3—Dy1—O13—C22	−104.9 (4)	O12—N8—C18—C17	158.2 (6)
N5—Dy1—O13—C22	−92.4 (4)	O12—N8—C18—C19	−19.9 (10)
O1—Dy1—N1—N2	72.0 (3)	O16—N9—C25—C24	31.5 (8)
O1—Dy1—N1—C43	−101.8 (4)	O15—N9—C25—C26	7.4 (7)
O2—Dy1—N1—N2	124.3 (3)	O15—N9—C25—C24	−172.6 (5)
O2—Dy1—N1—C43	−49.5 (4)	O16—N9—C25—C26	−148.5 (6)
O5—Dy1—N1—N2	−75.2 (3)	O20—N10—C32—C33	17 (7)
O5—Dy1—N1—C43	111.0 (4)	O19—N10—C32—C33	177 (5)
O9—Dy1—N1—N2	−155.2 (3)	O20—N10—C32—C31	−166 (5)
O9—Dy1—N1—C43	31.0 (4)	O19—N10—C32—C31	−5 (7)
O13—Dy1—N1—N2	158.3 (3)	O23—N11—C39—C38	−15.1 (14)
O13—Dy1—N1—C43	−15.5 (6)	O24—N11—C39—C38	167.6 (8)
N3—Dy1—N1—N2	−0.8 (3)	O24—N11—C39—C40	−12.5 (13)
N3—Dy1—N1—C43	−174.7 (5)	O23—N11—C39—C40	164.8 (11)
N5—Dy1—N1—N2	−1.7 (4)	O2—C1—C2—C7	173.5 (3)
N5—Dy1—N1—C43	−175.5 (4)	O1—C1—C2—C7	−4.2 (5)
O1—Dy1—N3—C46	−105.0 (3)	O2—C1—C2—C3	−3.9 (5)
O1—Dy1—N3—C50	83.1 (3)	O1—C1—C2—C3	178.4 (3)
O2—Dy1—N3—C46	−67.0 (3)	C1—C2—C7—C6	−177.5 (4)
O2—Dy1—N3—C50	121.1 (3)	C3—C2—C7—C6	−0.1 (6)
O5—Dy1—N3—C46	84.4 (3)	C7—C2—C3—C4	−0.7 (6)
O5—Dy1—N3—C50	−87.4 (3)	C1—C2—C3—C4	176.7 (3)
O9—Dy1—N3—C46	33.8 (3)	C2—C3—C4—C5	0.8 (6)
O9—Dy1—N3—C50	−138.0 (3)	C2—C3—C4—N6	−177.3 (4)
O13—Dy1—N3—C46	−171.6 (3)	N6—C4—C5—C6	178.0 (4)
O13—Dy1—N3—C50	16.5 (3)	C3—C4—C5—C6	−0.1 (6)
N1—Dy1—N3—C46	−4.4 (3)	C4—C5—C6—C7	−0.7 (7)
N1—Dy1—N3—C50	−176.3 (3)	C5—C6—C7—C2	0.8 (7)
N5—Dy1—N3—C46	174.8 (3)	O6—C8—C9—C14	178.3 (3)
N5—Dy1—N3—C50	3.0 (3)	O6—C8—C9—C10	−1.9 (4)
O1—Dy1—N5—N4	−79.5 (2)	O5—C8—C9—C10	176.8 (3)
O1—Dy1—N5—C53	91.4 (4)	O5—C8—C9—C14	−3.0 (5)
O2—Dy1—N5—N4	−101.1 (2)	C14—C9—C10—C11	−0.7 (5)
O2—Dy1—N5—C53	69.8 (4)	C8—C9—C14—C13	−179.9 (4)
O5—Dy1—N5—N4	75.8 (2)	C8—C9—C10—C11	179.5 (3)
O5—Dy1—N5—C53	−113.3 (4)	C10—C9—C14—C13	0.3 (6)
O9—Dy1—N5—N4	135.3 (2)	C9—C10—C11—N7	−178.7 (3)
O9—Dy1—N5—C53	−53.8 (4)	C9—C10—C11—C12	0.6 (6)
O13—Dy1—N5—N4	−168.7 (3)	C10—C11—C12—C13	−0.2 (7)
O13—Dy1—N5—C53	2.2 (4)	N7—C11—C12—C13	179.2 (4)
N1—Dy1—N5—N4	3.0 (3)	C11—C12—C13—C14	−0.2 (7)
N1—Dy1—N5—C53	173.9 (4)	C12—C13—C14—C9	0.2 (7)
N3—Dy1—N5—N4	2.1 (2)	O9—C15—C16—C17	177.0 (3)
N3—Dy1—N5—C53	173.0 (4)	O10—C15—C16—C21	165.1 (12)
O10—Dy2—O6—C8	−34.4 (5)	O9—C15—C16—C21	−5.3 (5)
O14—Dy2—O6—C8	−110.0 (6)	O10—C15—C16—C17	−12.6 (12)
O18—Dy2—O6—C8	−174.9 (3)	C21—C16—C17—C18	−3.1 (6)
O21—Dy2—O6—C8	−28.2 (4)	C17—C16—C21—C20	4.5 (6)
O17^i^—Dy2—O6—C8	47.8 (3)	C15—C16—C17—C18	174.6 (4)
O22^i^—Dy2—O6—C8	124.9 (3)	C15—C16—C21—C20	−173.1 (4)
O6—Dy2—O14—C22	92.0 (19)	C16—C17—C18—N8	−179.3 (5)
O10—Dy2—O14—C22	16.6 (18)	C16—C17—C18—C19	−1.3 (8)
O18—Dy2—O14—C22	−143 (2)	C17—C18—C19—C20	4.2 (9)
O21—Dy2—O14—C22	−65.7 (19)	N8—C18—C19—C20	−177.9 (6)
O17^i^—Dy2—O14—C22	1 (3)	C18—C19—C20—C21	−2.6 (9)
O22^i^—Dy2—O14—C22	160.0 (16)	C19—C20—C21—C16	−1.6 (8)
O6—Dy2—O18—C29	−142.6 (3)	O14—C22—C23—C28	−19.0 (12)
O10—Dy2—O18—C29	114.0 (7)	O14—C22—C23—C24	159.7 (11)
O14—Dy2—O18—C29	146.6 (7)	O13—C22—C23—C28	172.9 (3)
O21—Dy2—O18—C29	49.8 (4)	O13—C22—C23—C24	−8.4 (4)
O17^i^—Dy2—O18—C29	−17.6 (4)	C28—C23—C24—C25	1.9 (5)
O22^i^—Dy2—O18—C29	−81.7 (4)	C22—C23—C24—C25	−176.9 (3)
O6—Dy2—O21—C36	124.1 (4)	C24—C23—C28—C27	−1.7 (5)
O10—Dy2—O21—C36	130.1 (6)	C22—C23—C28—C27	177.1 (3)
O14—Dy2—O21—C36	−155.9 (7)	C23—C24—C25—C26	−0.5 (6)
O18—Dy2—O21—C36	−84.6 (4)	C23—C24—C25—N9	179.5 (4)
O17^i^—Dy2—O21—C36	44.6 (4)	C24—C25—C26—C27	−1.3 (6)
O22^i^—Dy2—O21—C36	−23.7 (5)	N9—C25—C26—C27	178.8 (4)
O6—Dy2—O17^i^—C29^i^	139.3 (5)	C25—C26—C27—C28	1.5 (6)
O10—Dy2—O17^i^—C29^i^	−147.4 (6)	C26—C27—C28—C23	0.0 (6)
O14—Dy2—O17^i^—C29^i^	−131.9 (15)	O18—C29—C30—C35	18.0 (5)
O18—Dy2—O17^i^—C29^i^	4.4 (5)	O17—C29—C30—C35	−161.0 (3)
O21—Dy2—O17^i^—C29^i^	−63.0 (5)	O17—C29—C30—C31	15.9 (5)
O6—Dy2—O22^i^—C36^i^	−157.9 (3)	O18—C29—C30—C31	−165.1 (3)
O10—Dy2—O22^i^—C36^i^	−123.4 (7)	C31—C30—C35—C34	−2.3 (6)
O14—Dy2—O22^i^—C36^i^	129.6 (7)	C35—C30—C31—C32	0.7 (6)
O18—Dy2—O22^i^—C36^i^	73.1 (3)	C29—C30—C31—C32	−176.3 (4)
O21—Dy2—O22^i^—C36^i^	9.9 (4)	C29—C30—C35—C34	174.6 (4)
Dy1—O1—C1—O2	−15.7 (3)	C30—C31—C32—C33	2.0 (7)
Dy1—O1—C1—C2	161.9 (3)	C30—C31—C32—N10	−175 (3)
Dy1—O2—C1—O1	15.5 (3)	C31—C32—C33—C34	−2.9 (8)
Dy1—O2—C1—C2	−162.1 (3)	N10—C32—C33—C34	174 (3)
Dy1—O5—C8—C9	155.8 (2)	C32—C33—C34—C35	1.2 (9)
Dy1—O5—C8—O6	−25.6 (5)	C33—C34—C35—C30	1.4 (8)
Dy2—O6—C8—C9	−80.8 (4)	O22—C36—C37—C42	2.3 (4)
Dy2—O6—C8—O5	100.6 (3)	O21—C36—C37—C38	2.6 (4)
Dy1—O9—C15—C16	174.5 (2)	O21—C36—C37—C42	−178.3 (3)
Dy1—O9—C15—O10	3.7 (12)	O22—C36—C37—C38	−176.8 (3)
Dy1—O13—C22—C23	−150.7 (3)	C36—C37—C38—C39	178.2 (3)
Dy1—O13—C22—O14	41.3 (13)	C42—C37—C38—C39	−0.9 (5)
Dy2—O14—C22—O13	−88.0 (17)	C38—C37—C42—C41	0.6 (6)
Dy2—O14—C22—C23	104.4 (17)	C36—C37—C42—C41	−178.5 (4)
Dy2^i^—O17—C29—C30	170.0 (3)	C37—C38—C39—N11	−179.0 (5)
Dy2^i^—O17—C29—O18	−8.9 (7)	C37—C38—C39—C40	1.1 (6)
Dy2—O18—C29—O17	19.0 (6)	N11—C39—C40—C41	179.0 (7)
Dy2—O18—C29—C30	−159.9 (2)	C38—C39—C40—C41	−1.1 (8)
Dy2—O21—C36—C37	−157.9 (3)	C39—C40—C41—C42	0.8 (8)
Dy2—O21—C36—O22	21.4 (6)	C40—C41—C42—C37	−0.6 (8)
Dy2^i^—O22—C36—C37	176.2 (2)	N1—C43—C44—C45	−0.2 (8)
Dy2^i^—O22—C36—O21	−3.1 (5)	C43—C44—C45—N2	1.2 (8)
C43—N1—N2—C46	−178.9 (4)	N2—C46—C47—C48	178.3 (5)
Dy1—N1—C43—C44	173.6 (4)	N3—C46—C47—C48	−2.3 (8)
N2—N1—C43—C44	−0.9 (6)	C46—C47—C48—C49	1.5 (8)
Dy1—N1—N2—C45	−173.7 (4)	C47—C48—C49—C50	0.0 (8)
Dy1—N1—N2—C46	5.8 (5)	C48—C49—C50—N3	−0.9 (7)
C43—N1—N2—C45	1.7 (6)	C48—C49—C50—N4	178.7 (4)
C46—N2—C45—C44	178.8 (5)	N4—C51—C52—C53	−0.1 (5)
N1—N2—C46—C47	170.0 (4)	C51—C52—C53—N5	0.3 (5)
C45—N2—C46—C47	−10.7 (8)		

Symmetry code: (i) −*x*, −*y*+1, −*z*.

### Hydrogen-bond geometry (Å, º)

**Table:**

*D*—H···*A*	*D*—H	H···*A*	*D*···*A*	*D*—H···*A*
C10—H10···O22^i^	0.93	2.45	3.366 (4)	170
C43—H43···O2^ii^	0.93	2.47	3.212 (5)	137
C51—H51···O6^iii^	0.93	2.32	3.243 (5)	172

Symmetry codes: (i) −*x*, −*y*+1, −*z*; (ii) −*x*+1, −*y*+2, −*z*+1; (iii) −*x*, −*y*+2, −*z*.
